# Common mental disorders among seasonal migrant farmworkers in Northwest Ethiopia

**DOI:** 10.1186/s12888-021-03068-7

**Published:** 2021-02-02

**Authors:** Kassahun Alemu Gelaye, Malede Mequanent Sisay, Temesgen Yihunie Akalu, Destaw Fetene Teshome, Haileab Fekadu Wolde, Getu Debalkie Demissie, Sintayehu Daba Wami, Telake Azale, Tadesse Awoke Ayele

**Affiliations:** 1 Department of Epidemiology and Biostatistics, Institute of Public Health, College of Medicine and Health Sciences, University of Gondar, Gondar, Ethiopia; 2 Department of Health Promotion and Behavioral Sciences, Institute of Public Health, College of Medicine and Health Science, University of Gondar, Gondar, Ethiopia; 3 Department of Environmental and Occupational Health and Safety, Institute of Public Health, College of Medicine and Health Sciences, University of Gondar, Gondar, Ethiopia

**Keywords:** Ethiopia, Farmers, Mental disorders, Social determinants of health

## Abstract

**Background:**

Seasonal migrant farmworkers in Ethiopia are a vulnerable segment of the population facing numerous threats to their mental health. This research aimed to determine the magnitude of common mental disorders (CMDs) and its associated factors among seasonal migrant farmworkers in the northwest of Ethiopia.

**Methods:**

A cross-sectional study was conducted. A total of 950 seasonal migrant farmworkers were selected randomly. CMDs were assessed using the self-reporting questionnaire (SRQ-20) and a structured questionnaire was employed to collect the associated characteristics of socio-demographic data. Data were analyzed using descriptive statistics, bivariate, and multivariable binary logistic regression. The adjusted odds ratio (AOR) with a 95% confidence level was used to declare a statistically significant association with CMDs.

**Results:**

The prevalence of CMDs was found to be 23.05% (219/950; 95% CI 20.47–25.84) among seasonal migrant farmworkers. The prevalence of psychological stress was 74.53% (708/950; 95% CI 71.65–77.20). Having a daily income below USD 5 (AOR = 1.53, 95% CI: 1.10–2.15), moderate perceived stress (AOR = 3.18, 95% CI: 1.18, 5.36), severe perceived stress (AOR = 16.15, 95% CI: 8.96, 29.11), and heat-related illness (AOR = 1.60, 95% CI: 1.11, 2.30) were associated with a higher likelihood of experiencing CMD. On the other hand, those seasonal migrant farmworkers who migrated for the first time (AOR = 0.38, 95% CI: 0.23–0.65) and those who received health related information (AOR = 0.60, 95% CI: 0.42, 0.85) were less likely to have CMDs.

**Conclusion:**

In this study, CMDs were found to be prevalent among seasonal migrant farmworkers. These findings highlight the importance of systematic development of community-based mental health services in combination with rural primary health care centers and an integrated approach to the health care of farmworkers such as screening, early identification, and treatment of CMDs of seasonal migrant farmworkers.

**Supplementary Information:**

The online version contains supplementary material available at 10.1186/s12888-021-03068-7.

## Background

Common mental disorders (CMDs) are the most prevalent mental disorders globally. CMDs include anxiety, depression, and somatic symptom disorders and are characterized by symptoms such as insomnia, fatigue, irritability, forgetfulness, difficulty concentrating, and somatic complaints [1, 2].

CMDs have been a neglected field in Africa [3–8]. Compared to other areas of health, mental health services are poorly developed, and often come last on the list of priorities for policymakers [9–12]. Between 2000 and 2015 the number of years lost to disability as a result of mental and substance use disorders increased by 52% [13]. In 2015, 17·9 million years were lost to disability as a consequence of CMDs [14]. In Ethiopia, the life expectancy of individuals with severe mental disorders is 30 years shorter than the general population. People living with mental or substance use disorders are more likely to become infected with HIV [15, 16]. Many of these earlier studies of mental health were focused on people attending health facilities as out-patients [17, 18]. Recently, governments, researchers, and journals have increased attention to mental health as the member states of the World Health Assembly committed to the Comprehensive Mental Health Action Plan [19]. In 2016, all the world’s governments included mental health in the Sustainable Development Goals: the third goal aimed to improve mental health and prevent and treat substance use disorders [20].

In Ethiopia, the agricultural sector encompasses a large variety of activities and demonstrates differences compared to other sectors in terms of health issues. Work in the open field, dependence upon working times and periods based on seasonal or climatic conditions, individuals conducting more than one job at a time, and agricultural fields being located outside of urban areas pose risks for farmworkers [21]. Farming is also associated with a range of physical and mental health risks because of the hard work under challenging conditions [22]. Psychological health disturbances have been shown to be more common in farmers and farm-workers [23]. Stress is associated with an increased prevalence of mental disorders, such as depression and anxiety [24].

Addressing the psychological needs of seasonal farmworkers in rural areas and the factors that influence the psychological state is essential [25, 26]. Poor mental health can be particularly difficult to identify, treat and overcome among agricultural workers facing multiple challenges, including psychological barriers, poor access to health services, and high rates of poverty [27, 28]. In western Kenya’s rural agricultural area,10.8% of agriculture workers experienced CMDs [29]. In Ethiopia, seasonal migrant farmworkers represent a population group that is often ignored and marginalized in society despite their active workforce status in agriculture. Studies on psychological disorders among farmers in the country had been rare until recently [6, 30]. To date, very limited research studies have been conducted that provide a picture of the mental health of Ethiopian seasonal migrant farmworkers. Previous epidemiological studies have documented and demonstrated increased mental health problems for the general population. In Ethiopia’s context, seasonal migrant farmworkers stay and work in rural areas, almost exclusively. Hence, it is important to understand the existence of CMDs in the sense of seasonal migrant farmworkers’ in rural areas.

To this end, the primary aim of this study was to estimate the prevalence of CMDs and to identify associated factors among seasonal migrant farmworkers in northwestern Ethiopia, and provide baseline evidence of mental health needs in the country for this specific population.

## Methods

### Study design and settings

A community-based cross-sectional study was conducted from February to March 2019 in the western Gondar region, Ethiopia. The West Gondar zone (including Metema and West Armachiho districts) is one of the largest extensive agricultural development corridors of Ethiopia which attract large populations of seasonal migrant farmworkers. These are fertile agricultural areas with large-scale cash crops such as sesame, maize, cotton, and sorghum. As a result, a hundred thousand migrants and seasonal farmworkers travel to these areas for work during weeding (July to August) and harvesting seasons (September to November). The area is endemic to malaria and visceral leishmaniasis. The overall temperature ranges from 22 °C to 43 °C. Except for some mountainous regions, almost all of the land in these districts is lowland.

### Study population

The study population was seasonal migrant farmworkers who migrated during agricultural seasons to these districts of the West Gondar zone during the data collection period. Seasonal migrant farmworkers are mainly engaged in cash crops such as sesame, cotton, but there is also substantial animal production. A seasonal migrant farmworker is an agricultural worker who is hired by a farm owned by someone else, usually hired on a part-time basis or a contract basis. Seasonal (non-migrant) farmworkers, on the other hand, earn a living in agriculture but remain in one location throughout the year. Migrants generally stay in the highlands of Ethiopia during the winter months and migrate northwest during planting or harvesting. The population of migrant farmworkers is ethnically diverse, with the majority stemming from the center of the Amhara region. Their ethnic composition therefore differs from that of this region of the country.

### Sampling procedure and sample size determination

The sample size was calculated using a population proportion formula. Since no similar study was conducted previously in Ethiopia, 50% was used to estimate the proportion of the population of farmworkers who experience a CMDs. The size of the sample was decided based on the following assumptions: we fixed the significance level at 95%, the error margin at 5%, and the power (1-β) at 80%. With a design effect of two, the total sample size needed was 885, assuming a 15% non-response rate. First, two kebeles were purposefully selected based on the size (Delelo in the Metema district and Abderafi in the western Armachiho district). In the first stage of sampling, primary sampling units (PSUs)(Camps) were formed by segmenting the farms so that each PSU would have approximately 460 migrant workers. Camps within study clusters have been identified using line listings from ongoing farms. There are 174 farms in Delelo and 560 in Abderafi. The typical number of seasonal migrant workers in each farm is estimated to be 460, making the total number of migrant workers 80,040 in 174 farms. Lastly, the population of each camp was listed as living and hired by farm owners. Subsequently, systematic sampling was used to determine the required sample from each study site.

### Data collection process

All data were collected through a questionnaire administered by the interviewer in Amharic. Interviewers participated in a 6-h training session and each completed practice interview before being approved to conduct study interviews. After informed consent was obtained from each participant, the interviewers worked in teams conducting one-on-one interviews with all camp residents who were interested in participating in the study. Interviews usually took place late in the afternoon or on the weekends when the workers were off duty at the safer place near the camps. No incentive was given to the participants. Regular supporting supervision was provided by the main investigators. Every day, the data were cross-checked for completeness and missing values by data collectors and supervisors.

### Variables of the study

A pretested and structured questionnaire was used to collect data. The tool was prepared first in English then translated into the local language (Amharic). Then it was re-translated to English by language experts to keep its consistency (Supplementary file 1). The questionnaire included demographic characteristics and other background information of the participants including sex, age, educational status, occupation before departure, family size, the residence of origin, marital status, average daily income, history of visits to farm areas, length of stay in months at farms, access to health information, sources of health information, working hours per day, working hours per week, heat stress and heat-related illness. The Self-Reported Questionnaire (SRQ-20) was used to evaluate the presence and prevalence of CMDs. The SRQ was originally designed by the WHO as a self-assessment scale but was also found to be suitable for interviewer-administration in settings of low literacy developing countries [31, 32]. The SRQ was cross culturally valid in Ethiopia among Amharic-speaking general populations; people in non-psychiatric treatment; and people in psychiatric treatment [33]. The optimal cut-off scores identified in previous studies were 4/5 and 6/7 for men and women, respectively. Each of the 20 items on the questionnaire receives a score of 0 or 1. A score of 1 indicates that the symptom was present during the last month before data collection; a score of 0 indicates that the symptom was absent. A cut-off point of 7/8 (7’yes’ a noncase, 8’yes’ a case) was used, which is the most commonly used cut-off point in developing countries [34]. It was used in previous community-based studies in Ethiopia [35]. The Cohen Perceived Stress Scale (PSS) was also used to assess perceived stress. The PSS, which is the most widely used psychological tool for measuring stress perception. The stress level is defined as a score of each item on a 5-point scale ranging from never (0) to almost always (4) indicating above 13 is considered to be psychological stress. The psychometric properties of the PSS has been tested in Ethiopian university students and found to be valid [36]. The PSS is a measure of the degree to which situations in one’s life are assessed as stressful. Items were designed to assess tap how unpredictable, uncontrollable, and overloaded respondents could find their lives. The scale also includes some direct queries about current levels of experienced stress ranging from 0 to 40 [37].

### Data processing and analysis

The data collected was encoded and entered into a computer using Epi-data version 3.1 programs and exported to STATA version 14. Descriptive statistics (frequency and percentages) were calculated and presented in the tables.

After performing assumption tests using crosstabs (chi-square), bivariate and multivariable binary logistic regression analyses were performed to examine the association of the outcome variable (CMD) with potential risk factors. These potential risk factors were selected a priori based on evidence from the existing literature and our theoretical assumptions that these factors would be relevant for CMD. Only factors that were associated with the CMD in the bivariate models were included in the corresponding multivariable model to limit the potential risk of over adjusting without compromising the identification of potential predictors for the outcome at a *p*-value of less than 0.25. Odds ratios with 95% CI were used to determine the strength of associations in the final regression model.

### Ethics approval and consent to participate

The protocol for the study was approved by the Ethical Review Committee of the College of Medicine and Health Sciences, University of Gondar. Moreover, we have received ethical approval from the Amhara Public Health Institute (APHI). Verbal informed consent was obtained from all respondents who were enrolled in the study. All participants have given written, informed consent to participate.

## Results

### Sociodemographic characteristics of respondents

A total of 950 adults participated in the study. The majority of respondents were male (943/950; 99.26%). The median (± IQR) age of participants was 25 years (IQR:20–29) with an age range of 13 to 67 years. About 98% (932/950) of respondents were Orthodox Christian followers. The majority of respondents (832; 87.58%) were rural residents. More than 25.58% (243) were married. About 42.74% (406) had primary education and 10.21% (97) had secondary and higher education. Approximately half of the respondents (56.63%) earned a daily income of ETB 120 (5 USD). Most (772; 81.26%) had migrated more than twice to the study region (Table 1).

**Table 1:** Sociodemographic and work-related characteristics of seasonal migrant farmworkers, northwest Ethiopia, 2019 (*n* = 950)

Characteristics	Categories	Number(%)
Sex	Male	943 (99.26%)
	Female	7 (0.74%)
Age (years)	Median (IQR): 25 (20–29)	
Educational Status	No education	447 (47.05%)
	Primary	406 (42.74%)
	Secondary and above	97 (10.21%)
Occupation before departure	Students	177 (18.63%)
	Farmers	645 (67.89%)
	Others*(housemaid, merchant, unemployed, etc.)	128 (13.47%)
Family size	1–2	88 (9.26%)
	3–5	536 (56.42%)
	6+	326 (34.32%)
Residence of origin	Rural	832 (87.58%)
	Urban	118 (12.42%)
Religion	Orthodox Christianity	932 (98.11%)
	Muslim	15 (1.58%)
	Others (protestant, Catholic)	3 (0.32%)
Marital status	Single	649 (68.32%)
	Married	243 (25.58%)
	Divorced	58 (6.11%)
Average daily income	< 5 USD	412 (43.37%)
	≥ 5 USD	538 (56.63%)
History of visits in the area	once	178 (18.74%)
	2+	772 (81.26%)
Length of stay in months	≤1	540 (56.84%)
	> 1	410 (43.16%)
Access to health information	Yes	566 (59.58%)
	No	384 (40.42%)
Source of information, mass media	Yes	328 (34.53%)
	No	622 (65.47%)
Source of information, health worker	yes	509 (53.64%)
	no	440 (46.36%)
Source of information, friends/family	Yes	270 (28.48%)
	No	678 (71.52%)
Source of information, poster/notice	yes	90 (9.49%)
	No	858 (90.51%)
Source of information, school	Yes	145 (15.31%)
	No	802 (84.69%)
Working hours per day	≤ 8	259 (27.26%)
	8+	691 (72.74%)
Working hours per week	≤ 48	97 (10.21%)
	48+	853 (89.79%)
Heat stress	Yes	757 (79.68%)
	No	193 (20.32%)
Heat-related illness	Yes	572 (60.21%)
	No	378 (39.79%)
Ever incident in this working environment	Yes	291 (30.63%)
	No	659 (69.37%)
CMDs	Yes	219 (23.05%)
	No	731 (76.95%)
Perceived stress	Yes	708 (74.53%)
	No	242 (25.47%)

NB: IQR means interquartile ranges between the first and third quartile of the data. CMD was measured using SRQ and perceived stress using PSS

### Work and access of information related characteristics of respondents

Nearly 60% of respondents (566) had access to health information from different sources. Mass media (328; 34.53%) and health workers (509; 53.64%) were common sources of health information. Approximately 72.74% (691) of respondents worked more than 8 h a day in the last month. Around 79.68% (747) of the respondents suffered from heat stress. Overall, 60.21% (572)) were affected by heat-related illness (Table 1).

### Prevalence of CMD

The total prevalence of CMD and perceived stress was found to be 23.05% (219/950) [95% CI: 20.47–25.84] and 74.53% (731/950) [95% CI: 71.65–77.20], respectively. About 25.47% (242/950) of respondents reported mild stress, 58.95% (560/950) reported moderate stress and 15.58% (148/950) had extreme stress (Table 1).

### Factors associated with CMD

Multivariate logistic regression analyses revealed that perceived stress levels, average daily income, receipt of health information, heat-related illness, and longer farm stays were significantly associated with CMDs.

The likelihood of having CMD were 1.53 times (AOR = 1.53, 95% CI: 1.10–2.15) higher for those with daily income below USD 5.00 than for those with daily income above USD 5.00. Respondents with moderate perceived stress (AOR = 3.18, 95% CI:1.18, 5.36) and severe stress (AOR = 16.15, 95% CI:8.96, 29.11) were more likely to experience CMDs than those with seasonal farmworkers suffering from heat-related illnesses were 1.6 times more likely to have CMDs than those who did not have a heat-related illness (AOR = 1.60, 95% CI: 1.11, 2.30).

Those seasonal migrant farmworkers who came to the farm for the first time were 62% less likely to experience mental health problems than those who came to the farm more than twice (AOR = 0.38, 95% CI:0.23–0.65). Seasonal workers with health-related information were 40% less likely to have CMDs than those without information (AOR = 0.60, 95% CI: 0.42, 0.85) (Table 2).

**Table 2:** Logistic regression analysis of seasonal migrant farmworkers, northwest Ethiopia, 2019 (*n* = 950)

Characteristics	Categories	CMD		COR (95% CI)	AOR (95% CI)
		Yes	No		
Educational Status	Educated	124	379	1.00	1.00
	No education	95	352	0.83 (0.61, 1.12)	0.81 (0.57,1.16)
Residence	Urban	35	83	1.00	1.00
	Rural	184	648	0.67 (0.44, 1.03)	0.72 (0.44,1.19)
Marital status	Married	64	179	1.00	1.00
	Single	145	504	0.81 (0.57, 1.13)	0.97 (0.66,1.45)
	Divorced	10	48	0.58 (0.28, 1.22)	0.82 (0.37,1.83)
Average daily income	≥ 5 USD	105	433	1.00	1.00
	< 5 USD	114	298	1.58 (1.17, 2.14)	1.53 (1.10,2.15) *
History of visits in the area	2+	198	574	1.00	1.00
	New	21	157	0.39 (0.24, 0.63)	0.38 (0.23, 0.65) *
Stress status	No	19	223	1.00	1.00
	Moderate	113	447	2.97 (1.78, 4.95)	3.18 (1.89,5.36) *
	Severe	87	61	16.74 (9.45,29.64)	16.2 (8.96,29.11)*
Heat-related illness	No	160	319	1.00	1.00
	Yes	16	412	2.10 (1.51, 2.93)	1.60 (1.11,2.30) *
Access to health information	No	451	280	1.00	1.00
	Yes	115	104	0.68 (0.51, 0.93)	0.60 (0.42, 0.85) *

NB: *COR* crude odds ration, *AOR* adjusted odds ration, *CI* Confidence interval; * is *p*-value < 0.05

## Discussion

Our results suggest that seasonal migrant farmworkers in northwestern Ethiopia experienced substantial levels of CMDs symptoms. Almost a quarter of participants suffered from CMD, and more than 75% of people reported stress at some stage of their working life. This finding is consistent with the findings done from Ethiopian immigrants who were non-farmworkers [15, 35, 38, 39], which found that 21.58%. The similarity might reflect the challenging nature of the work and the work environment that increases the psycho-social stresses on seasonal migrant farmworkers. However, the few earlier studies were mostly performed from out-patient clinic attendees, which is relatively higher than reported in Ethiopia [15–17, 30, 38, 40]. The current study might reflect a lack of quality mental health services in rural areas.

Additionally, seasonal migrant farmworkers may be more likely to use substances, alcohol, and other illicit drugs as a means of coping with their marginalized status and the stigma and discrimination they face. In particular, rural areas, where there is a large amount of cash crop production, attract large numbers of workers results in the burden of mental health disorders. The Ethiopian Government has shown its commitment to the expansion of mental health care, but particular farm workers have remained in the shadows – i.e. excluded from mental health care [8, 41]. This indicates that the health of farmers has been neglected in their mental health.

The prevalence of CMD reported here is consistent with reports from other LLMICs [26, 42] and global studies [1, 43]. For example, a study from 26 high-income countries and 37 low and middle-income counties found a prevalence of 17.6% within the past 12 months and 29.2% across their lifetime among the adults’ population [43]. This may be because, in many countries, civil conflicts with alcohol, tobacco, and drug-related problems are becoming increasingly common, and are associated with adverse effects on people’s mental health and well-being, particularly stress disorders. However, this study is contradictory to studies conducted in Kenya [29]. This discrepancy could be due to the use of various tools to assess CMDs, SRQ-20 used for this study. In addition, the difference in the study population characteristics and dynamics could be the possible reason for the discrepancy.

In our study, the average daily income was strongly associated with a CMD that has found a vicious cycle of poverty and mental illness [4, 26, 44]. This could be a lack of income for a livelihood that could lead to stressful and unsafe situations that might trigger CMDs People living in poverty face difficulties in meeting basic needs, have poor access to treatment and face challenges in participating in paid labour.

Similarly, perceived stress had a statistically significant association with CMD. The high prevalence could be explained by a high level of stress and suffering faced by farmers due to a higher burden of social and household responsibilities [45]. Similarly, factors such as the experience of insecurity and hopelessness, rapid social change, and the risks of violence and physical ill-health may explain the greater vulnerability of those living in poverty to CMDs [26]. An earlier study indicated that high levels and prolonged durations of perceived stress were often accompanied by symptoms of depression and/or anxiety [46]. Contrarily to this, the previous history of visits and exposure to health education were protective against CMDs in our study. Those who have lived in the area for a long time may have a better coping mechanism and therefore are less likely to have CMDs than those who have lived for a short time. They might also benefit from better support networks if they have lived there for a longer time. A study in India has indicated that higher education might mitigate the association between elevated stress and CMD [47]. However, in this study, education had no relationship with CMD. Future efforts to improve the mental health of seasonal migrant farmworkers should focus on preventing CMD. Clinical outreach programs to promote better mental health for seasonal migrant farmworkers should be implemented in rural health care. Our study shows that the mental health of seasonal migrant farmworkers remains poorly understood, in particular the personal and social factors that may affect the health of farmworkers. The results of this study provide some insights for health workers, service providers, and researchers working to protect the mental health of farmworkers. The results obtained in this study should be evaluated with caution due to the possible limitations of a cross-sectional study. Selection bias might be a problem, as we only include and evaluate those who were working at farms during data collection. Recall bias and social desirability bias might be introduced and limit the reliability of study findings. Furthermore, uncertainty regarding the previous history of psychiatric disorder over a lifetime may be a further limitation.

## Conclusions

Findings from this study suggest that CMDs remain a significant public health problem among seasonal migrant workers. These findings highlight the importance of systematic development of community-based mental health services in combination with rural primary health care centers and an integrated approach to the healthcare of farmworkers such as screening, early identification, and treatment of mental health problems of seasonal migrant farmworkers. Further in-depth research is strongly recommended for a better understanding of the promotion of psychological wellbeing in Ethiopia.

## Supplementary Information

**Additional file 1: Supplementary file 1**. English version survey questionnaire.

## Data Availability

All data are included in the manuscript. However, the data upon which the result is based could be accessed at a reasonable request.

## Abbreviations

AOR: Adjusted Odds Ratio
COR: Crude Odds Ratio
SRQ: Self-reporting Questionnaire
CI: Confidence Interval
CMD: common mental disorders
PSS: Perceived Stress Scale
