# Supplementary material for: Common mental disorders among seasonal migrant farmworkers in Northwest Ethiopia
Source: BMC Psychiatry. 2021 Feb 2;21:69. doi: 10.1186/s12888-021-03068-7 (PMC7852114; doi:10.1186/s12888-021-03068-7)
Supplement: Supplementary file 1 — Additional file 1: Supplementary file 1. English version survey questionnaire. [file 12888_2021_3068_MOESM1_ESM.docx]

**English Version Questionaire**

Code_____________________________

**Permanent residence**

Regional State: __________________Zone:________________District: _______________

**Farming area**

District: __________________Company name:________________

**Part I: Socio demographic characteristics**

| S.No | | Questions | Response | |
| --- | --- | --- | --- | --- |
|  | | Sex | 1. Male 2. Female | |
|  | | Age (in years) | ------------- | |
|  | | What is the highest education level you completed?   1. Unable to read and write 2. Able to read and write without formal education | 1. 1-4 Grades 2. 5-8 Grades 3. 9-10 Grades | 1. 11-12 Grades 2. Diploma/above |
|  |  | Main occupation before departure   1. Student 2. Farmers 3. Housemaid 2. Merchant 5. Unemployed 6. Others (specify) ______ |  |  |
|  |  | Family size (in number) | ______ |  |
|  |  | Residence 1. Rural 2. Urban |  |  |
|  |  | Ethnicity 1.Amhara 2. Kimant 3. Tigre 4. Others (Specify) ___ |  |  |
|  |  | What is your religion? 1. Orthodox 2. Muslim 3. Protestant 4. Catholic 5. Other (specify)_____________ |  |  |
|  |  | Marital status 1. Single 2. Married 3. Divorced 4. Widowed 5. Separated |  |  |
|  |  | Salary | _________ETB(**Daily**) |  |
|  |  | How many times have you been here? | ______times |  |
|  |  | How long have you been living here in the current visit? | _____Days |  |

| S.No | Questions | Response | Remark |
| --- | --- | --- | --- |
|  | Have you seen or heard any health information messages from any source recently? | 1. Yes, 2. No |  |
|  | What was the source of health information? |  |  |
|  | - 1. Mass media (radio/ TV) | 1. Yes, 2. No |  |
|  | - 1. Health workers | 1. Yes, 2. No |  |
|  | - 1. Friends/Family | 1. Yes, 2. No |  |
|  | - 1. Posters/notices/magazines/newspaper | 1. Yes, 2. No |  |
|  | - 1. School | 1. Yes, 2. No |  |
|  | - 1. Others(specify) | _____________________ |  |

**Part II. Source of information about health**

**Part III. Source of information about health**

| **Heat stress assessment, the incidence of heatstroke, heat exhaustion, and heat cramps** | | | |
| --- | --- | --- | --- |
| **Heat stress assessment** | | | |
|  | Does the air feel warm or hot? | 1. Yes 2. No |  |
|  | Is there a radiant heat source present eg the sun, furnaces; ovens; kiln walls, kilns; dryers; hot surfaces & machinery, exothermic chemical reactions, molten metals, etc.) | 1. Yes 2. No |  |
|  | Is there any equipment that produces steam? | 1. Yes 2. No |  |
|  | Is the workplace affected by external weather conditions? | 1. Yes 2. No |  |
|  | Do you wear PPE that is vapor impermeable? | 1. Yes 2. No |  |
|  | Do you complain that the air is humid? | 1. Yes 2. No |  |
|  | Is warm or hot air blowing onto you | 1. Yes 2. No |  |
|  | Is the work-rate moderate to intensive? | 1. Yes 2. No |  |
|  | Is PPE being worn to protect against harmful chemicals, asbestos, flames, extreme heat, etc? | 1. Yes 2. No |  |
|  | Is respiratory protection being worn? | 1. Yes 2. No |  |
|  | Do you think that heat stress is a problem? | 1. Yes 2. No |  |
|  | Do you complain of feeling warm or hot? | 1. Yes 2. No |  |
| **Incidence of heatstroke, heat exhaustion, and heat cramps** | | | |
|  | Have you had the following sign and symptoms (heat stroke)   1. Chills 1.Yes 2.No 2. 2.Restlessness 1.Yes 2.No 3. Irritability 1.Yes 2.No 4. Unconscious 1.Yes 2.No 5. Hot, dry skin 1.Yes 2.No | |  |
|  | Have you had the following sign and symptoms (Heat exhaustion)   1. Fatigue 1.Yes 2.No 2. Weakness 1.Yes 2.No 3. Blurred vision 1.Yes 2.No 4. Dizziness, 1.Yes 2.No 5. Headache 1.Yes 2.No 6. Profuse sweating 1.Yes 2.No | |  |

**Part IV. Mental health-related**

1. **Level of mental health**

| **SNo** | **SRQ item**   \|  \| \| --- \| | **1.Yes** | **2.No** | **Remark** |
| --- | --- | --- | --- | --- | --- |
|  | Do you often have a headache? |  |  |  |
|  | Is your appetite poor? |  |  |  |
|  | Do you sleep badly? |  |  |  |
|  | Are you easily frightened? |  |  |  |
|  | Do your hands shake? |  |  |  |
|  | Do you feel nervous, tense, or worried? |  |  |  |
|  | Is your digestion poor? |  |  |  |
|  | Do you have trouble thinking clearly? |  |  |  |
|  | Do you feel unhappy? |  |  |  |
|  | Do you cry more than usual? |  |  |  |
|  | Do you find it difficult to enjoy your daily activities? |  |  |  |
|  | Do you find it difficult to make decisions? |  |  |  |
|  | Is your daily work suffering? |  |  |  |
|  | Are you unable to play a useful part in life? |  |  |  |
|  | Have you lost interest in things? |  |  |  |
|  | Do you feel that you are a worthless person? |  |  |  |
|  | Has the thought of ending your life been on your mind |  |  |  |
|  | Do you feel tired all the time? |  |  |  |
|  | Are you easily tired |  |  |  |
|  | Do you have uncomfortable feelings in your stomach? |  |  |  |

**B. Level of Stress**

| **S.No** | **item** | **0.N** | **1.AN** | **2.S** | **3.FO** | **4.VO** |
| --- | --- | --- | --- | --- | --- | --- |
|  | In the past month, how often have you been upset because of something that happened  Unexpectedly? |  |  |  |  |  |
|  | In the past month, how often have you felt unable to control the important things in your life? |  |  |  |  |  |
|  | In the past month, how often have you felt nervous or stressed? |  |  |  |  |  |
|  | In the past month, how often have you felt confident about your ability to handle personal  Problems? |  |  |  |  |  |
|  | In the past month, how often have you felt that things were going your way? |  |  |  |  |  |
|  | In the past month, how often have you found that you could not cope with all the things you had to do? |  |  |  |  |  |
|  | In the past month, how often have you been able to control irritations in your life? |  |  |  |  |  |
|  | In the past month, how often  have you felt that you were on top of things? |  |  |  |  |  |
|  | In the past month, how often have you been angry because of things that happened that were outside of your control? |  |  |  |  |  |
|  | In the past month, how often have you felt that difficulties were piling up so high that you could not overcome them? |  |  |  |  |  |

N=Never; AN=Almost-never; S=Sometimes; FO=Fairly-often; VO=Very-often
